# CD3^+^RUNX3^+^ lymphocyte density; an independent prognostic factor in colon and lung adenocarcinoma but not in lung squamous cell carcinoma

**DOI:** 10.1038/s41598-026-38765-4

**Published:** 2026-02-05

**Authors:** Thomas K. Kilvaer, Dagny Førde, Erna-Elise Paulsen, Mona Irene Pedersen, Ana Paola Lombardi, Mehrdad Rakaee, Hallgeir Selven, Tom Donnem, Lill-Tove Rasmussen Busund, Sigve Andersen

**Affiliations:** 1 Department of Oncology, University Hospital of North Norway, Tromso, Norway; 2 Department of Clinical Medicine, UiT The Arctic University of Norway, Tromso, Norway; 3 Department of Pulmonary Medicine, University Hospital of North Norway, Tromso, Norway; 4 Department of Medical Biology, UiT The Arctic University of Norway, Tromso, Norway; 5 Department of Clinical Pathology, University Hospital of North Norway, Tromso, Norway

**Keywords:** NSCLC, Lung cancer, Colon cancer, CRC, RUNX3, CD3, TNM-I, Biomarker, Digital pathology, Biomarkers, Cancer, Immunology, Oncology

## Abstract

RUNX3, an important regulator of T-cell differentiation plays a crucial role in activation and maintenance of various T-cells including cytotoxic-, helper- and memory-cells. Former studies have highlighted RUNX3 as a prognostic factor across several cancer types, but few have studied its cell-type specific expression patterns. We used multiplex immunohistochemistry to assess and quantify cells co-expressing CD3 and RUNX3 in three cohorts consisting of 452 colon adenocarcinoma (COAD), 239 lung adenocarcinoma (LUAD) and 307 lung squamous cell carcinoma (LUSC) patients. Further, we correlated the expression of CD3/RUNX3 to disease-specific survival and to previously investigated immune markers in these cohorts. We found that a high density of CD3^+^RUNX3^+^ cells was an independent prognostic factor for disease-specific survival in COAD (HR 0.37, 95% CI 0.21–0.64) and LUAD (HR 0.61, 95% CI (0.39–0.97), but not in LUSC. Interestingly, RUNX3 expression was positively correlated with CD8 in all cohorts, CD20 in LUAD and FOXP3 in LUSC. Univariate subgroup analyses revealed that COAD patients with high numbers of both CD3^+^RUNX3^+^ and CD8^+^ cells rarely experienced a DSS event (HR 0.24, 95% CI 0.15–0.39). Contrasting previous studies, we did not observe RUNX3 expression in epithelial cells. A high level of CD3^+^RUNX3^+^ density is an independent prognostic factor in COAD and LUAD, but not in LUSC. In COAD, a subset of patients in stage II/III with CD3^+^RUNX3^+^_high_/CD8 ^+^ _high_ may be spared adjuvant treatment due to excellent prognosis. However, further studies are needed to confirm and elucidate the protective role of CD3^+^RUNX3^+^ cells in COAD and LUAD.

**Supplementary Information:**

The online version contains supplementary material available at 10.1038/s41598-026-38765-4.

## Introduction

 Lung- and colorectal cancer are the number one and three most commonly occurring cancers and rank as the first and second leading causes of cancer related deaths worldwide^1^. In 2022 alone, approximately 4,400,000 people were diagnosed with lung- or colorectal cancer, resulting in around 2,700,000 fatalities^1^. Lung- and colorectal cancer share some characteristics including their epithelial origin. However, they arise in organs specifically adapted to their respective functions, are exposed to distinct environments and are host to vastly different microbiomes. Lung cancer is categorized into small-cell lung cancer (15–20%) and non-small lung cancer (NSCLC 80–85%). NSCLC is further subdivided into the histological subgroups adenocarcinoma (LUAD) and squamous cell carcinoma (LUSC) of which the former now comprises nearly 50% of cases^2^. Of particular interest, both colorectal cancer (CRC) and NSCLC are targeted by the adaptive immune system. In fact, different types of immune infiltration have proven as robust biomarkers for patient survival both in CRC and NSCLC^3–5^. This is reflected in current treatment guidelines and research focus. For early stage colon cancer, the ESMO guidelines endorses Immunoscore^®^ in conjunction with TNM scoring in certain circumstances^6^, while our group drives an initiative to implement an immune cell score as a supplement to the TNM staging system in NSCLC^7^. Further, in the metastatic setting, tumor infiltrating lymphocyte (TIL) density predict response to immune check point inhibitors (ICIs) in NSCLC^8,9^.

RUNX3 is part of the runt related transcription factor (RUNX) family and is considered a master regulator potentially involved in many important intra-cellular signaling pathways including transforming growth factor-β, Wnt, Hedgehog and Notch^10^. In mammalian development, RUNX3 is involved in central nervous system maturation and in CD4 repression during CD8^+^ lymphocyte lineage determination from double CD4^+^CD8^+^ precursor lymphocytes in the thymus^11,12^. In carcinogenesis, RUNX3 has been proposed as a significant regulator in several types of cancer including CRC and NSCLC^10,13–15^. Further, and supporting its role as a tumor suppressor in CRC and NSCLC, increased expression of RUNX3 in epithelial cells has been identified as a positive prognostic factor in many studies^16–19^. Intriguingly, and contradicting the role of RUNX3 as a direct suppressor of epithelial cancers, a critical review by Lotem et al. based on data from whole genome studies, concludes that RUNX3 is predominantly expressed in immune- and not in epithelial cells^20^. In cancer immunity, RUNX3 is one of many transcription factors that drives the formation of CD8^+^ tissue resident memory cells (CD8^+^T_RM_) – pivotal to mounting an efficient anti-tumor response^21,22^. Of particular interest for CRC, RUNX3 regulates lymphocytes in the gut epithelium and is partly responsible for forming double positive CD4^+^CD8^+^ lymphocytes in this location^23^.

In our previous work on colon cancer (COAD), we found that a high density of RUNX3^+^ cells was an independent prognosticator of favorable disease-specific survival (DSS)^24^. While we did distinguish between expression in the tumor and stromal compartments, we did not differentiate between expression in different cell-types. However, we noted that the majority of RUNX3 positive cells morphologically resembled lymphocytes.

In the current study we explore RUNX3 as a prognostic biomarker in three cohorts of COAD, LUAD and LUSC patients treated with curative intent surgery. Based on lessons learned from our previous study in COAD^24^, we hypothesize that RUNX3 is predominantly expressed in T-lymphocytes and that its prognostic impact is associated with its expression in immune cells. Our analysis pipeline, including multiplex IHC, digital pathology and supervised machine-learning-assisted assessment of marker expression, enables us to look at the prognostic impact of both the overall density of RUNX3 positive cells and the density of specific phenotypes according to CD3 status. Further, we aim to elucidate the correlations between RUNX3 and other immune related markers previously investigated in the cohorts.

## Materials and methods

### Patients and clinical samples

Details regarding the inclusion of patients and collection of clinical data have been reported previously^25,26^. *In brief*, the COAD, LUAD and LUSC cohorts comprises 452, 239 and 307 patients who underwent curative intent surgery for their primary disease in Northern Norway. The COAD patients were treated from 1998 to 2007 with a median follow-up of survivors of 173 months (range 119–239), while the LUAD and LUSC patients were treated from 1990 to 2010 with a median follow-up of survivors of 82 (range 33–267) and 88 (range 35–250) months, respectively.

### Tissue microarray construction

All pathological specimens were collected from the archives of the regional departments of pathology located at the University Hospital of Northern Norway and Nordland Central Hospital. The diagnostic slides were reviewed by experienced pathologists. In this process, relevant areas were identified on hematoxylin and eosin (H&E) stained slides and subsequently transferred to the corresponding tumor tissue blocks ensuring correct sampling for tissue microarray (TMA) construction. A total of four TMA cores, two from tumor and two from adjacent stroma, were sampled for each patient and inserted into recipient TMA blocks. A total of ten and eleven TMA blocks were constructed for the COAD and NSCLC cohorts, respectively. Our TMA construction pipeline is extensively documented in previous publications^25–27^.

### Immunohistochemistry

The double-stain immunohistochemistry (IHC) procedure was conducted using the Discovery Ultra Research instrument (Roche, 05987750001) and antibodies and reagents validated for IHC. The new Roche high quality *Next-Generation chromogen technology for multiplexing* was used in this study. Protocol optimization, including the incorporation of appropriate controls and selection of relevant tissues, was performed *in-house*. The antibodies utilized in this study were RUNX3 (ab135248, Abcam) and CD3 (2GV6, Ventana). Prior to antibody detection, tissue sections underwent heating, deparaffinization, and antigen retrieval. Blocking was incorporated to prevent unspecific antibody bindings to other tissue structure. The procedure included primary antibody application, blocking, addition of secondary multimer antibodies, amplification, and detection. To avoid cross-reactivity of antibodies and enzymes between first and second staining sequence a heat deactivation step was mandatory. Green and Purple chromogens were used to visualize RUNX3 and CD3, respectively. Nuclei were counterstained with hematoxylin. Finally, the sections were washed, dehydrated, and mounted for analysis. Details of the optimized protocols are presented in Table S1, and a comprehensive list of reagents and products is provided in Table S2.

### Digital quantification of marker expressions

All TMAs were digitized using a 3DHistech Pannoramic Flash III slide scanner (3DHistech Ltd. Budapest Hungary) and processed in QuPath v0.5.0 (The University of Edinburgh, Scotland)^28^. Cell detection was conducted using a StarDist model with weights trained on the hematoxylin channel of the Lizard dataset^29^. To accurately capture the RUNX3 positive cells, the model was applied to a composite image channel comprising the maximum value of the color deconvolved hematoxylin or RUNX3 image channels. Intensity values were then calculated for each cell and a RandomTrees cell classifier was trained to distinguish CD3^+^RUNX3^−^, CD3^+^RUNX3^+^, CD3^−^RUNX3^+^ and CD3^−^RUNX3^−^ cells. To prevent necrotic cells and areas with excessive anthracosis to influence results, these were also included in the classifier for NSCLC. A sample script that can be used to emulate our workflow in QuPath is included in the supplementary material. Following classification, the mean densities of overall CD3 and RUNX3 positive cells and subgroups according to CD3 and RUNX3 status were exported to R for further analyses.

### Statistical methods

All statistical analyses were conducted in RStudio, version 2023.12.1 Build 402 and R version 4.3.1 with the packages “cowplot”, “ggplot2”, “gtable”, “grid”, “greidExtra”, “Hmisc” and “reshape2”. Missing data for the investigated markers were considered random events (loss of TMA cores, lack of tumor in TMA cores and other artifacts introduced during the staining process precluding scoring all TMA cores for a given patient) and hence data were not imputed.

We used χ^2^ and Fisher’s exact tests to investigate the relations between investigated markers and clinicopathological variables. Spearman’s rank correlations were used to test the correlations both between investigated markers and between these markers and previously tested immune cell markers in the different cohorts.

To test the investigated marker’s potential as prognostic biomarkers we conducted univariable log-rank-tests and visualized the corresponding relationships between low vs. high expression and survival using the Kaplan-Meier method. Survival was defined as the time between the registration of the surgical specimen at the department of pathology to death caused by the disease (disease-specific survival, DSS). The optimal cut-point maximizing the difference between survival curves, but still ensuring that no derived groups contained less than 25% of patients was used. Further, to test the prognostic value of the markers in the presence of clinicopathological variables, Cox regression models were constructed. All significant clinicopathological variables from univariate analyses were considered for inclusion in the multivariable models. The proportional hazards assumption for each model was tested using Schoenfeld residuals (data not shown). In case of a positive Shoenfeld test, the models were retested with follow-up restricted to 5 years after diagnosis. Testing for linearity was not deemed necessary since none of the models incorporated continuous variables.

The significance threshold for all tests was set at *p* ≤ 0.05. Since we consider the current work hypothesis generating, no formal correction of multiple testing was conducted.

### Ethical considerations

The study was conducted according to the guidelines of the Declaration of Helsinki, and was approved by the Regional Committee for Medical and Health Research Ethics North (REK Nord, protocol IDs: 2011/2503 and 2011/2151). The need for informed consent was waived by REK Nord due to the retrospective nature of the study. The reporting of clinicopathological variables, survival data and biomarker expression was conducted in accordance with the REMARK guidelines^30^.

## Results

### Clinicopathological variables

Clinicopathological variables for the included cohorts are extensively documented in previous publications and for convenience summarized in S3 Table ^25,26^. In brief, COAD patients were older at the time of diagnosis (median age COAD 76, LUAD 65 and LUSC 68), more likely to be female (COAD 54%, LUAD 42% and LUSC 25%), experienced a larger degree of severe weightloss (COAD 20%, LUAD 8% and LUSC 11%) and were less likely to experience a DSS event during follow-up (COAD 24%, LUAD 47% and LUSC 36%).

### Distribution of CD3^+/−^RUNX3^+/−^ their patterns of expression and associations with clinicopathological variables

**Fig. 1:**
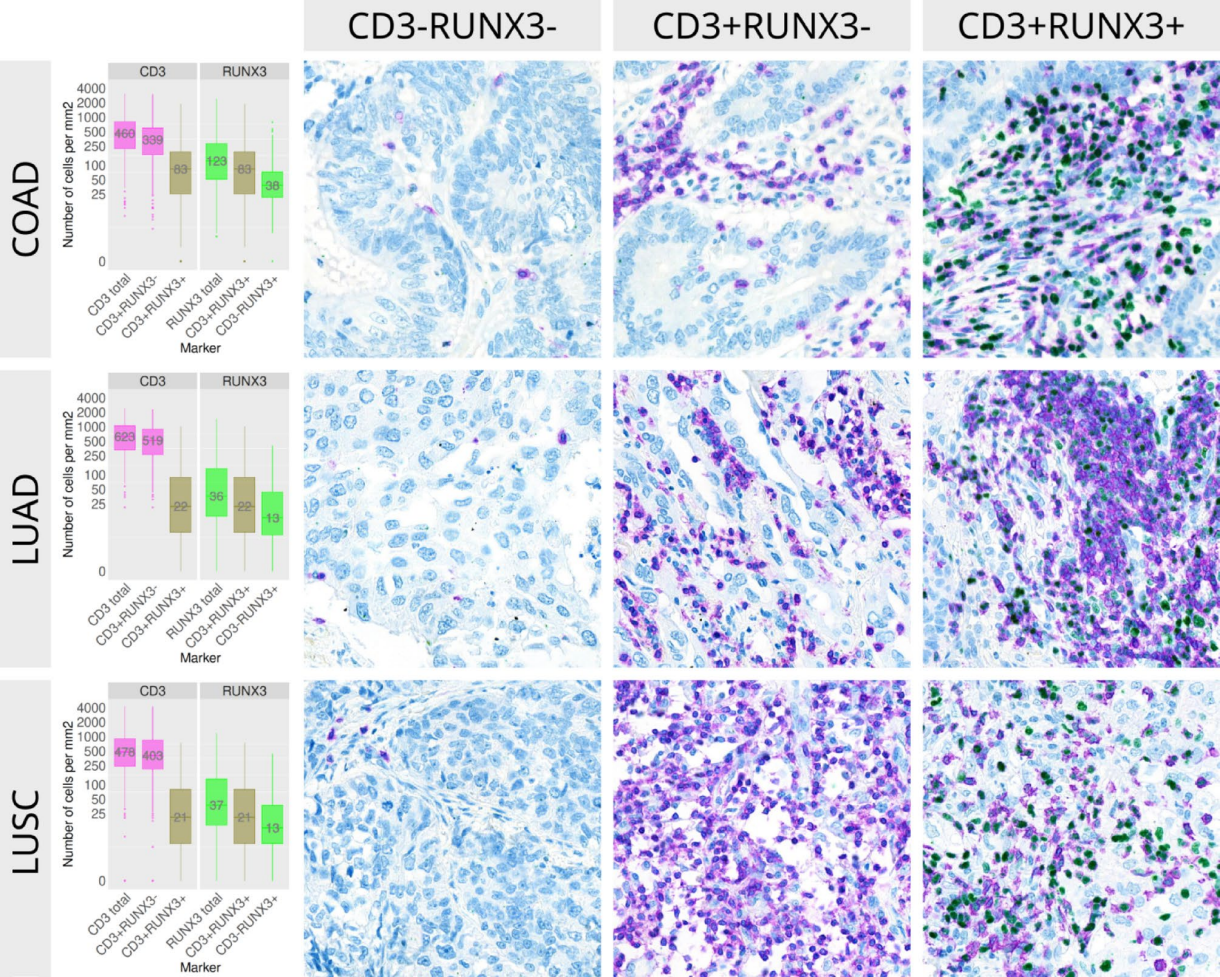
The distribution (col 1) and patterns of expression (cols 2–4) of CD3 and RUNX3 co-expression* in COAD (row 1), LUAD (row 2) and LUSC (row 3). *CD3^-^RUNX3^+^ were nearly always present alongside a strong and combined CD3^+^RUNX3^-^/CD3^+^RUNX3^+^ infiltrate.

Figure 1 summarizes the overall distribution of CD3^+/−^RUNX3^+/−^ cells across the cohorts and give examples of high and low infiltration of the different cell subtypes according to CD3^+/−^/RUNX3^+/−^ status. As expected, both CD3 and RUNX3 was expressed on lymphocytes. Interestingly, we observed no RUNX3 expression in epithelial cells across all cohorts. Further, the number as well as distribution of CD3 + and RUNX3^+^ cells varied across different tissue compartments and between patients. The median density of CD3^+^RUNX3^+/−^ cells was higher in LUAD (~ 600 cells/mm^2^) compared to COAD and LUSC (~ 450 cells/mm^2^), while the median density of CD3^+/−^RUNX3 ^+^ cells was higher in COAD (~ 125 vs. ~ 35 cells/mm^2^). Further, both CD3 ^+^ RUNX3^+^ and CD3 ^−^ RUNX3^+^ cells were more abundant in COAD, compared to LUAD and LUSC. In addition, we noticed that a strong infiltration of CD3^−^RUNX3^+^ cells was almost always accompanied by at least an equal amount of CD3^+^RUNX3^+/−^ cells. However, in a handful of patients across cohorts, CD3^−^RUNX3^+^ cells were predominant. Notably, epithelial cells did not express RUNX3.

Tables S4-6 summarizes the associations between dichotomized cell densities and clinicopathological variables for COAD, LUAD and LUSC respectively. For all cohorts, high densities of CD3^−^RUNX3^+^ cells were associated or tended to be associated with female gender (*P* = 0.015, 0.072 and 0.029, for COAD, LUAD and LUSC respectively). For COAD patients, high densities of CD3^+^RUNX3^+^ and CD3^−^RUNX3^+^ cells were associated with tumors of the right colon while a high density of CD3^+^RUNX3^−^ cells was associated with pathological stage (pStage) I and II. For LUAD patients, a high overall CD3^+^ cell density was associated with lower tStage and pStage. While for LUSC patients, a high density of CD3^−^RUNX3^+^ cells was associated with T1 tumors.

### Correlations with previously investigated immune markers

Correlations with immune markers previously investigated in the cohorts are presented in Figures S1-3. Of note, a substantial number of immune markers were available for analyses in LUAD and LUSC, while only CD8 was available for COAD. Only correlations with a r value < 0.3 are considered of clinical interest.

For all three cohorts, overall RUNX3, CD3^−^RUNX3^+^ and CD3^+^RUNX3^+^ and overall CD3, CD3^+^RUNX3^−^ and CD3^+^RUNX3^+/−^ were moderately to strongly correlated (*r* > 0.5–0.6). Interestingly, CD3^+^RUNX3^−^ was not correlated with CD3^−^RUNX3^+^ and only moderately to very weakly correlated with CD3^+^RUNX3^+^ COAD, *r* = 0.49; LUAD, *r* = 0.15);LUSC, *r* = 0.23). In COAD, CD3^+^RUNX3^+^ (*r* = 0.49) and CD3^−^RUNX3^+^ (*r* = 0.24) were moderately and very weakly correlated with CD8. In LUAD, CD3^+^RUNX3^+^ was weakly correlated with CD20 (*r* = 0.32), CD8 (*r* = 0.34) and CD3 (*r* = 0.35). And in LUSC, CD3^+^RUNX^+^ was weakly correlated with FOXP3 (*r* = 0.35), CD8 (*r* = 0.37) and CD3 (*r* = 0.38) while CD3^−^RUNX3^+^ was correlated with FOXP3 (*r* = 0.31).

### Univariate analyses

**Fig. 2:**
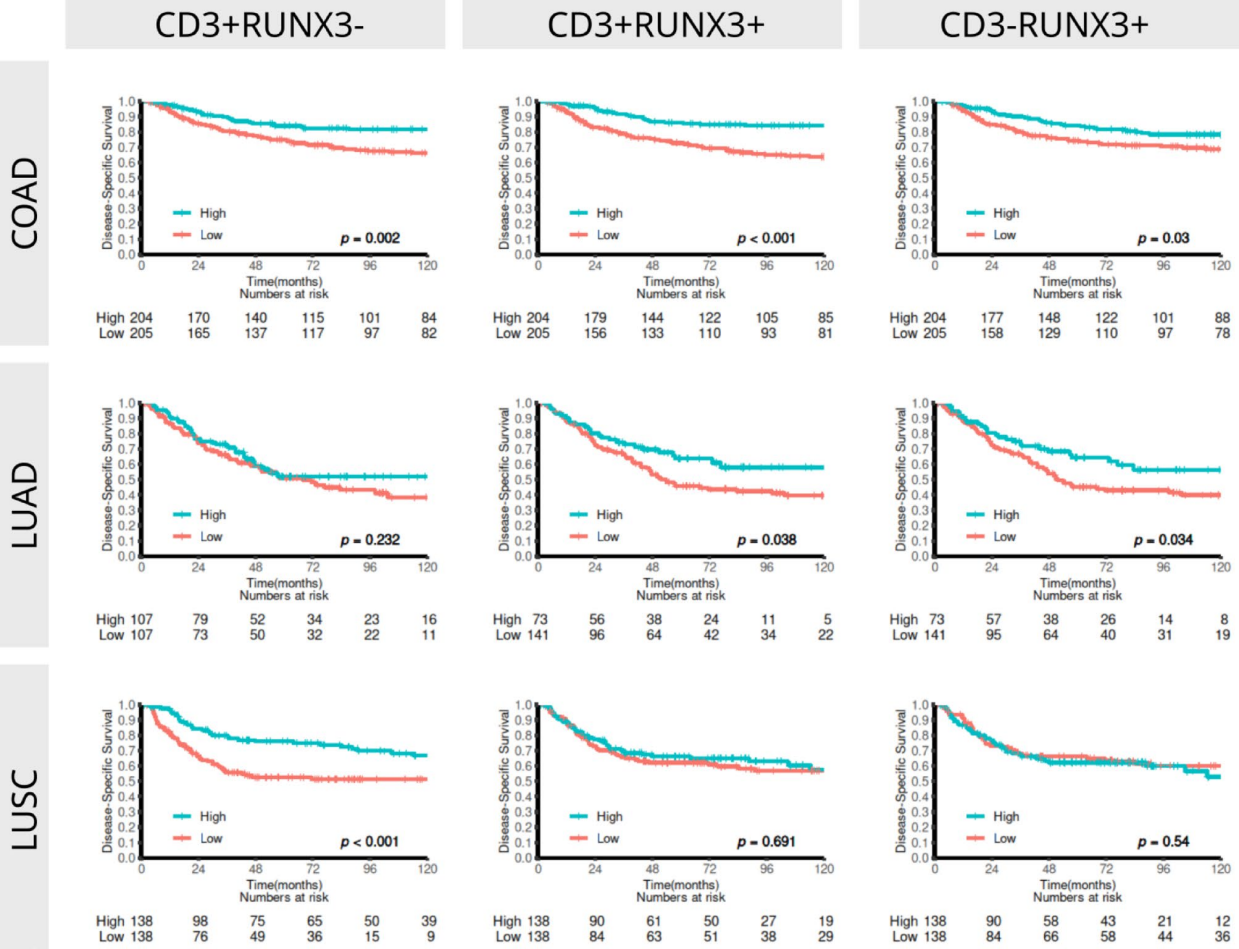
Disease-specific survival curves for different combinations of CD3 and RUNX3 densities in COAD (*n* = 452), LUAD (*n* = 239) and LUSC (*n* = 307). OAD, colon adenocarcinoma; LUAD, lung adenocarcinoma; LUSC, lung squamous cell carcinoma.

Univariate survival analyses of different combinations of CD3 and RUNX3 densities as prognosticators of DSS in COAD, LUAD and LUSC patients are summarized in Table 1; Fig. 2 and Figure S4. The prognostic impact of clinicopathological variables as predictors of DSS have previously been reported for all three cohorts and is included as reference in S3 Table. *In brief*, increasing pStage, poorly differentiated tumors and vascular infiltration, were clinicopathological predictors of adverse DSS in all three cohorts. In addition, for COAD patients increasing age and severe weightloss and for LUAD patients male sex, were also associated with poor DSS. As expected, overall CD3 density was a strong prognosticator of DSS in COAD (*p* < 0.001) and LUSC (*p* < 0.001) and near significant in LUAD (*p* = 0.054). Overall RUNX3 density was a prognosticator of DSS in COAD (*p* < 0.001) and in LUAD (*p* = 0.042), but not in LUSC. When looking at cell subtypes, CD3^+^RUNX3^−^ cell density was a significant prognosticator of DSS in COAD (*p* = 0.002) and LUSC (*p* < 0.001), but not in LUAD, while CD3^+^RUNX3^+^ and CD3^−^RUNX3^+^ densities were prognosticators of DSS in COAD (*p* < 0.001 and 0.030) and LUAD (*p* = 0.038 and 0.034).

**Table 1:** Univariate analyses, of different combinations of CD3 and RUNX3 density as prognosticators of DSS in COAD, LUAD and LUSC patients (log-rank test test, n = 452, 239 and 307).

	COAD					LUAD					LUSC				
	*N*(%)	5 Y	Med	HR(95%CI)	*P*	*N*(%)	5 Y	Med	HR(95%CI)	*P*	*N*(%)	5 Y	Med	HR(95%CI)	*P*
CD3+					< 0.001					0.054					< 0.001
Low	205(45)	73	NA	1		107(45)	47	57	1		138(45)	51	71	1	
High	204(45)	86	NA	0.41(0.28–0.61)		107(45)	56	NA	0.68(0.46–1.01)		138(45)	78	235	0.43(0.29–0.65)	
Missing	43(10)					25(10)					31(10)				
RUNX3+					< 0.001					0.042					0.864
Low	205(45)	73	NA	1		107(45)	44	52	1		138(45)	64	235	1	
High	204(45)	86	NA	0.5(0.34–0.75)		107(45)	59	NA	0.67(0.45–0.99)		138(45)	64	NA	1.04(0.69–1.55)	
Missing	43(10)					25(10)					31(10)				
CD3 + RUNX3-					0.002					0.232					< 0.001
Low	205(45)	75	NA	1		107(45)	51	68	1		138(45)	53	NA	1	
High	204(45)	84	NA	0.53(0.35–0.79)		107(45)	52	NA	0.79(0.54–1.16)		138(45)	76	235	0.5(0.33–0.75)	
Missing	43(10)			+		25(10)					31(10)				
CD3 + RUNX3+					< 0.001					0.038					0.691
Low	205(45)	73	NA	1		141(59)	46	52	1		138(45)	62	235	1	
High	204(45)	86	NA	0.41(0.28–0.62)		73(31)	64	NA	0.63(0.42–0.94)		138(45)	66	NA	0.92(0.62–1.38)	
Missing	43(10)					25(10)					31(10)				
CD3-RUNX3+					0.030					0.034					0.540
Low	205(45)	74	NA	1		141(59)	45	52	1		138(45)	66	235	1	
High	204(45)	84	NA	0.64(0.43–0.96)		73(31)	64	NA	0.62(0.42–0.93)		138(45)	62	127	1.13(0.76–1.69)	
Missing	43(10)					25(10)					31(10)				
CD3 + RUNX3+/ CD8					< 0.001					0.211					0.013
-/-	133(29)	72	NA	1		78(33)	48	57	1		88(29)	56	NA	1	
+/-	64(14)	73	NA	0.76(0.41–1.41)		25(10)	59	NA	0.63(0.34–1.18)		50(16)	53	64	1.16(0.63–2.14)	
-/+	71(16)	74	NA	0.86(0.47–1.57)		58(24)	42	47	0.95(0.58–1.55)		48(16)	79	235	0.48(0.26–0.85)	
+/+	139(31)	92	NA	0.24(0.15–0.39)		48(20)	66	NA	0.59(0.35-1)		86(28)	73	NA	0.58(0.35–0.96)	
Missing	45(10)					30(13)					35(11)				

COAD, colon adenocarcinoma; LUAD, lung adenocarcinoma; LUSC, lung squamous cell carcinoma.

### Multivariable analyses

Multivariable models of different combinations of CD3 and RUNX3 are summarized in Table 2. Based on the results from the univariate analyses, separate models for overall and cell subtype specific density were made for COAD and LUAD, while for LUSC only an overall model was made. For COAD, age, pStage, CD3 (HR 0.58 95% CI 0.36–0.95) and RUNX3 (HR 0.62, 95% CI 0.38-1) and age, pStage, CD3^+^RUNX3^+^ (HR 0.37, 95% CI 0.21–0.64) were significant prognosticators of DSS in the overall and cell subtype specific models. For LUAD, gender, ECOG, pStage and differentiation and gender, ECOG, differentiation, pStage, CD3^+^RUNX3^+^ (HR 0.61, 95% CI 0.39–0.97) and CD3^−^RUNX3^+^ (HR 0.63, 95% CI 0.4–0.98) were retained in the overall and cell type specific models, respectively. For LUSC, ECOG, pStage, differentiation and overall CD3 (HR 0.41, 95% CI 0.26–0.64) were retained in the overall model.

**Table 2:** Multivariable models of different combinations CD3 and RUNX3 density as prognostic markers of DSS in COAD, LUAD and LUSC patients and including relevant clinicopathological variables (Cox’s proportional hazards test, *n* = 452, 239 and 307).

	COAD				LUAD				LUSC	
	Overall		Cell subtypes		Overall		Cell subtypes		Overall	
	HR (95% CI)	P	HR (95% CI)	P	HR (95% CI)	P	HR (95% CI)	P	HR (95% CI)	P
Age	1.03(1.01–1.05)	0.002	1.04(1.02–1.06)	< 0.001						
Gender										
Female					1.000		1.000			
Male					1.48(0.97–2.24)	0.067	1.51(1-2.29)	0.049		
ECOG										
1					1.000		1.000		1.000	
2					1.33(0.88–2.01)	0.181	1.44(0.94–2.18)	0.091	1.62(1.06–2.48)	0.027
3					2.35(0.99–5.59)	0.052	2.59(1.09–6.19)	0.032	1.47(0.61–3.51)	0.388
pStage										
1	1.000		1.000		1.000		1.000		1.000	
2	2.04(0.79–5.26)	0.142	2.15(0.83–5.57)	0.113	1.71(1.05–2.8)	0.032	1.76(1.08–2.87)	0.024	1.47(0.87–2.5)	0.150
3	7.73(3.1-19.29)	0.000	8.05(3.23–20.08)	0.000	3.16(1.91–5.22)	0.000	3.19(1.94–5.25)	< 0.001	4.51(2.67–7.59)	< 0.001
Differentiation										
Well					1.000		1.000		1.000	
Moderate					1.91(0.97–3.75)	0.060	1.92(0.98–3.76)	0.056	1.47(0.62–3.49)	0.383
Poor					2.02(1.05–3.86)	0.034	2(1.05–3.83)	0.036	2.21(0.94–5.21)	0.069
CD3 + overall										
Low	1.000				1.000				1.000	
High	0.58(0.36–0.95)	0.031			0.85(0.57–1.28)	0.442			0.41(0.26–0.64)	< 0.001
RUNX3 + overall										
Low	1.000				1.000				1.000	
High	0.62(0.38-1)	0.050			0.74(0.49–1.11)	0.144			1.4(0.92–2.12)	0.118
CD3 + RUNX3-										
Low			1.000							
High			0.78(0.5–1.22)	0.277						
CD3 + RUNX3+										
Low			1.000				1.000			
High			0.37(0.21–0.64)	< 0.001			0.61(0.39–0.97)	0.036		
CD3-RUNX3+										
Low			1.000				1.000*			
High			1.26(0.77–2.08)	0.360			0.63(0.4–0.98)	0.040		

*Numbers from separate model.

Abbreviations: COAD, colon adenocarcinoma; LUAD, lung adenocarcinoma; LUSC, lung squamous cell carcinoma.

### Co-expressions between CD3 + RUNX3+ and CD8

**Fig. 3:**
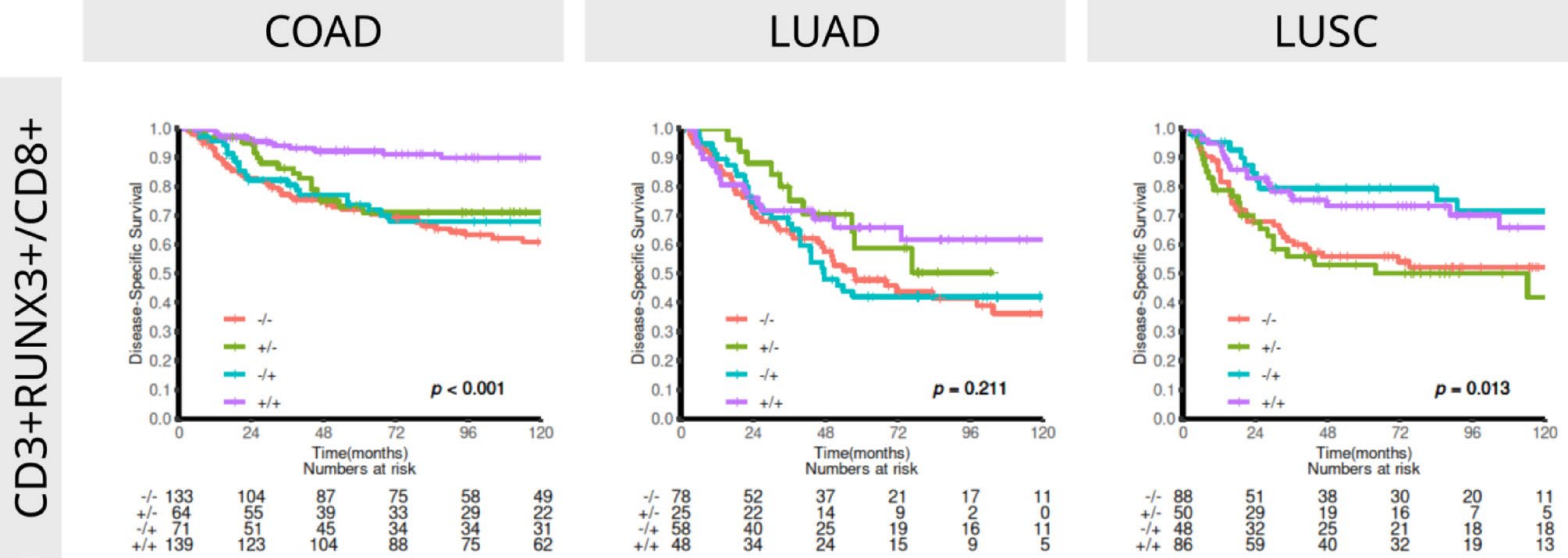
Disease-specific survival curves for different combinations of CD3^+^RUNX3^+^ and CD8 in COAD (*n* = 452), LUAD (*n* = 239) and LUSC (*n* = 307).

Based on our survival analyses and the moderate to strong correlations observed between CD3^+^RUNX3^+^ and CD8 across cohorts, co-expression analyses of CD3^+^RUNX3^+^ densities and CD8 was warranted. The univariate co-expression analyses for all cohorts are presented in Table 1; Fig. 3. Interestingly, the co-expression revealed a subgroup of CD3^+^RUNX3^+^_high_/CD8^+^ _high_ COAD patients with exceptional survival (multivariate HR 0.23 95% CI 0.12–0.39). Of particular interest, only 3/67 and 9/44 COAD patients in pStages II and III with CD3^+^RUNX3^+^_high_/CD8^+^ _high_ experienced a DSS event, compared to 16/63 and 29/56 in the CD3^+^RUNX3^+^_low_/CD8^+^ _low_ group. For LUSC, the co-expression was significant, but upon inspection it was not caused by the co-expression per se, but driven by CD8 alone (Fig. 3).

## Discussion

### Summary of results

To our knowledge this is the first study to investigate cell-type specific expression of RUNX3 in COAD, LUAD and LUSC. We conducted our experiments on three large cohorts of COAD, LUAD and LUSC patients and employed next-generation chromogen technology for multiplexing IHC and supervised machine-learning-assisted analyzes in QuPath to assess and quantify various subsets of CD3^+/^RUNX3^+/−^ cells. Importantly, RUNX3 expression was detected in immune cells and not in epithelial cells (Fig. 1).

Our results demonstrate CD3^+^RUNX3^+^ positive cell density as an independent prognosticator for DSS in COAD (HR 0.37, 95% CI 0.21–0.64) and LUAD (HR 0.61, 95% CI 0.39–0.97), but not in LUSC (Table 2). Moreover, capitalizing on previous studies of immune cell markers in the investigated cohorts, we found moderate to strong correlations between CD3^+^RUNX3^+^ expression and CD8 for all cohorts, CD20 for LUAD and FOXP3 for LUSC (Figures S1-3). Finally, co-expression analyses of CD3^+^RUNX3^+^ and CD8, revealed a strong and potentially synergistic prognostic signal in COAD (univariate HR 0.24 95% CI 0.15–0.39), while it did not reach significance in LUAD and was driven by CD8 alone in LUSC (Fig. 3; Table 2).

### Expression of CD3 and RUNX3

In a previous study, we showed that RUNX3 is a potential prognostic biomarker for early stage COAD patients^24^. Although, while we did distinguish between RUNX3^+^ cells in the stromal vs. tumor compartment, we were not able to differentiate cell-type specific expression. However, the morphologic features of cells expressing RUNX3 suggested a lymphocyte phenotype. In the current work, we observe that in COAD, LUAD, and LUSC, the majority of RUNX3^+^ cells also express the pan lymphocyte marker CD3. And further, that single positive RUNX3^+^ cells mostly occur alongside a strong infiltration of CD3^+^RUNX3^+^ cells. In our previous study we used DAB to identify RUNX3^+^ cells and observed significant background staining in mucus and in the cytoplasm of epithelial cells. In the current study we utilize multiplex IHC with advanced high quality IHC chromogens and antibody blocking to give more specific staining (Fig. 1). Leveraging these modern techniques, we observe no RUNX3 staining in epithelial cells – in neither nuclei nor cytoplasm. Our findings coincide with the conclusion of a comprehensive review by Lotem et al. that critically appraises the evidence of RUNX3 as a tumor suppressor in epithelial cancers. They conclude that: (1) epithelial tissue does not express RUNX3 and (2) RUNX3 indirectly suppresses tumor growth by driving an efficient anti-tumor immune response^20^. Moreover, we observe that the density of RUNX3^+^ cells is four times higher in COAD compared to LUAD and LUSC, thus indicating a more prominent role in this entity. Our results suggest that RUNX3 is unlikely to exert a direct epithelial cell–intrinsic role in established COAD, LUAD, or LUSC tumors. This contrasts previous report of RUNX3 expression in epithelial cells^16–18^. However, these studies did not evaluate RUNX3 in the presence of a phenotype marker like CD3 (pan T-cells) or CK. As a consequence, their results may reflect an immune-cell derived signal. This underscores the importance of a cell-resolved approach when evaluating biomarkers expressed in multiple lineages.

### The prognostic role of RUNX3 in COAD and LUAD

The prognostic role of RUNX3 expression in COAD and NSCLC is explored in several former studies^16–18,31,32^,of which the majority focus on RUNX3 expression in epithelial cells/the epithelial compartment or do not distinguish between cell types/compartments. Several of them describe a global RUNX3 expression in normal tissue and a loss of/variable RUNX3 expression in tumor tissues. Surprisingly, as discussed in the previous paragraph, we did not observe RUNX3 expression in epithelial/cancer cells in either of our three large cohorts comprised of nearly ~ 1000 COAD, LUAD, and LUSC patients. However, similar to most previous studies in COAD and LUAD, we found that a high density of RUNX3^+^ cells is a positive prognostic marker. Moreover, our multiplex IHC pipeline enables us to identify CD3^+^RUNX3^+^ density as the main driver behind RUNX3 as a prognosticator in COAD and LUAD. As far as we know, our study is the first to report this finding. Our results align well with studies that report immune cell infiltration as a positive prognostic factor in COAD and LUAD and is further supported by the fact that RUNX3 is one of the key drivers of CD8^+^ Tissue resident memory cells (T_RM_) development – a known prerequisite for an efficient adaptive immune response^3–5,21^. Additionally, in COAD, RUNX3 is linked to the formation of double positive CD4^+^CD8^+^ lymphocytes with special regulatory functions in the gut epithelium, which can possibly explain the strong prognostic impact of CD3^+^RUNX3^+^ in this entity^23^.

### RUNX3 lacks prognostic value in LUSC

In contrast to adenocarcinoma histology (COAD and LUAD), CD3⁺RUNX3⁺ cell density was not associated with outcome in LUSC. This divergence likely reflects known heterogeneity in the tumor immune microenvironment between LUAD and LUSC, with systematic differences in immune cell composition and functional states across histological subtypes. Single-cell transcriptomic and immune profiling studies have demonstrated that immune landscapes differ substantially across NSCLC histological subtypes. In particular, these analyses suggest that LUSC tumors may be relatively enriched for regulatory and exhausted T-cell populations and other immunosuppressive features of the tumor microenvironment, which could attenuate the prognostic impact of specific T-cell subsets^33,34^. Together, these factors may help explain why RUNX3-expressing T cells do not confer the same prognostic information in LUSC as observed in adenocarcinomas.

### Potential clinical impact of RUNX3 in COAD and LUAD

We observe excellent prognoses for patients with pTNM stage I COAD in our cohort. Of these patients only 5/70 succumbed to their disease and no discernible prognostic value of RUNX3 was observed. However, in stages II and III, only 9 vs. 22% and 26 vs. 55% experienced a DSS event in the high vs. low CD3^+^RUNX3^+^ groups respectively (data not shown). Consequently, our results indicate that for stage II COAD patients, CD3^+^RUNX3^+^ status may be used to steer adjuvant chemotherapy as patients in the high group already exhibit exceptional prognoses and risk limited benefit of further and potentially harmful treatment. For stage III patients, our results are less clear. However, it is possible to envision a use case where CD3^+^RUNX3^+^_low_ patients are selected for intensified adjuvant treatment due to their less than stellar prognosis. In LUAD the main prognostic value of CD3^+^RUNX3^+^ was for patients in pTNM stage I where only 11% in the high vs. 48% in the low group experienced a DSS event (data not shown). For pTNM stage II and III no obvious prognostic value of CD3 ^+^ RUNX3^+^ was observed (data not shown). While pTNM stage I LUAD patients generally are spared additional treatment, adjuvant treatment for patients with CD3 ^+^ RUNX3^+^_low_ may be considered. In LUAD, further investigations should try to elucidate the dismal prognosis we observe in stage II and III CD3 ^+^ RUNX3^+^_high_ patients.

### Limitations

Several limitations of this study should be acknowledged. First, its retrospective design precludes causal inference and, limits the ability to assess functional mechanisms underlying the observed associations. Further, even though surgery still is the main curative treatment for the included patients adjuvant treatment have evolved significantly. The latter may alter the outcome for some patients that previously succumbed to their disease and thus change the impact of the investigated biomarkers in contemporary cohorts. Second, key molecular and microenvironmental variables, including but likely not limited to MSI/MMR status, KRAS/BRAF mutations and tumor budding in colorectal cancer, and STK11/KEAP1 alterations, KRAS/ALK/ROS1/SMARC4 and further details on the extent of smoking exposure in lung cancer, were not uniformly available and could not be incorporated into multivariable models. Third, although the cohorts are large and well annotated, they originate from a single geographic region with limited ethnic and cultural diversity. This may reduce the validity of our results in other populations. Fourth, around ~ 10% of patients had insufficient tumor tissue in the TMAs for biomarker evaluation. Although we consider TMA core loss, lack of tumor tissue in TMA cores and other tissue damage random events, we cannot rule out the possibility that our data may harbor a hidden systematic error accountable for part of the missing samples. Fifth, we believe the methods we use are well documented and reproducible in principle. Nevertheless, we know that slight changes in tissue handling and storage influence staining, and consequently also subsequent steps in our pipeline like cell detection and classification. We stress that the current protocol is fine tuned to our historic samples. Thus, it seems likely that either adjustments of the protocol to local practice or a rigorous standardization of tissue handling from surgery through staining and scanning is warranted before application in another center or in a clinical study. Sixth, since we regard our study as hypothesis generating, we have not applied any correction for multiple testing. This may lead to false positive results, especially in the correlation analyses (Figures S1-3). However, we did apply a rather stringent interpretation of the correlation results that may in part mitigate som of this effect. Further, we did observe nearly global correlation between the investigated markers and distinct groups of immune markers that partly validates our approach.

## Conclusion

The current study expands on our previous work in COAD, and adds important insights into the role of RUNX3 in COAD, LUAD and LUSC. We demonstrate that RUNX3 is predominantly expressed in immune cells and that CD3^+^RUNX3^+^ cell density is a strong and promising prognostic factor in COAD and LUAD, but not in LUSC. In addition, co-expression analyses of CD3^+^RUNX3^+^ and CD8 reveals one third of COAD patients in our cohort who have a 92% chance to be disease-free after 5 years. These results are of particular interest because they imply that a significant percentage of COAD patients are without need of adjuvant therapy. For the future, our results imply that studies should focus on the co-expression of an epithelial marker like CK, with RUNX3 and CD8 and/or CD4 or other specific immune cell markers in COAD and/or LUAD and try to understand the lack of prognostic signal for RUNX3 in LUSC.

## Supplementary Information

Below is the link to the electronic supplementary material.

Supplementary Material 1

## Data Availability

The data presented in this study are available upon reasonable request to the corresponding author. The data are not publicly available due to privacy regulations.

## Abbreviations

CD: Cluster of differentiation
CRC: Colorectal cancer
COAD: Colon adenocarcinoma
DSS: Disease-specific survival
H&E: Hematoxylin and eosin
IHC: Immunohistochemistry
LUAD: Lung adenocarcinoma
LUSC: Lung squamous cell carcinoma
ML: Machine learning
NSCLC: Non-small cell lung cancer
nStage: Node stage
pStage: Pathological stage
REK: Regional committee for medical and health research ethics
REMARK: Reporting recommendations for tumour MARKer prognostic studies
RUNX: Runt related transcription factors
TMA: Tissue microarray
TNM: Tumor node metastasis
tStage: Tumor stage

## References

[1] Bray, F. et al. Global cancer statistics 2022: GLOBOCAN estimates of incidence and mortality worldwide for 36 cancers in 185 countries. *CA Cancer J. Clin.* **74**, 229–263 (2024). 38572751 10.3322/caac.21834

[2] Houston, K. A., Mitchell, K. A., King, J., White, A. & Ryan, B. M. Histologic lung cancer incidence rates and trends vary by Race/Ethnicity and residential County. *J. Thorac. Oncol.* **13**, 497–509 (2018). 29360512 10.1016/j.jtho.2017.12.010 PMC5884169

[3] Kilvaer, T. K. et al. Digitally quantified CD8 + cells: the best candidate marker for an immune cell score in non-small cell lung cancer? *Carcinogenesis* **41**, 1671–1681 (2020). 33035322 10.1093/carcin/bgaa105 PMC7791621

[4] Donnem, T. et al. Stromal CD8 + T-cell Density—A promising supplement to TNM staging in Non–Small cell lung cancer. *Clin. Cancer Res.* **21**, 2635–2643 (2015). 25680376 10.1158/1078-0432.CCR-14-1905

[5] Pagès, F. et al. International validation of the consensus immunoscore for the classification of colon cancer: a prognostic and accuracy study. *Lancet* **391**, 2128–2139 (2018). 29754777 10.1016/S0140-6736(18)30789-X

[6] Argilés, G. et al. Localised colon cancer: ESMO clinical practice guidelines for diagnosis, treatment and follow-up. *Ann. Oncol.* **31**, 1291–1305 (2020). 32702383 10.1016/j.annonc.2020.06.022

[7] Donnem, T. et al. Strategies for clinical implementation of TNM-Immunoscore in resected nonsmall-cell lung cancer. *Ann. Oncol. Off J. Eur. Soc. Med. Oncol.* **27**, 225–232 (2016). 10.1093/annonc/mdv560 26578726

[8] De Lopez, M. et al. Role of tumor infiltrating lymphocytes and Spatial immune heterogeneity in sensitivity to PD-1 axis blockers in non-small cell lung cancer. *J. Immunother Cancer*. **10**, e004440 (2022). 35649657 10.1136/jitc-2021-004440 PMC9161072

[9] Rakaee, M. et al. Machine learning-based immune phenotypes correlate with STK11/KEAP1 co-mutations and prognosis in resectable NSCLC: a sub-study of the TNM-I trial. *Ann. Oncol.* **34**, 578–588 (2023). 37100205 10.1016/j.annonc.2023.04.005

[10] Ito, Y., Bae, S. C. & Chuang, L. S. H. The RUNX family: developmental regulators in cancer. *Nat. Rev. Cancer*. **15**, 81–95 (2015). 25592647 10.1038/nrc3877

[11] Taniuchi, I. et al. Differential requirements for runx proteins in CD4 repression and epigenetic Silencing during T lymphocyte development. *Cell* **111**, 621–633 (2002). 12464175 10.1016/s0092-8674(02)01111-x

[12] Inoue, K. et al. Runx3 controls the axonal projection of proprioceptive dorsal root ganglion neurons. *Nat. Neurosci.* **5**, 946–954 (2002). 12352981 10.1038/nn925

[13] Liang, Y., He, L., Yuan, H., Jin, Y. & Yao, Y. Association between RUNX3 promoter methylation and non-small cell lung cancer: a meta-analysis. *J. Thorac. Dis.* **6**, 694–705 (2014). 24976992 10.3978/j.issn.2072-1439.2014.04.09 PMC4073407

[14] Shin, E. J. et al. Epigenetic inactivation of RUNX3 in colorectal cancer. *Ann. Surg. Treat. Res.* **94**, 19–25 (2018). 29333422 10.4174/astr.2018.94.1.19 PMC5765274

[15] Yu, G. P. et al. Application of RUNX3 gene promoter methylation in the diagnosis of non-small cell lung cancer. *Oncol. Lett.* **3**, 159–162 (2012). 22740873 10.3892/ol.2011.450 PMC3362545

[16] Araki, K. et al. Expression of RUNX3 protein in human lung adenocarcinoma: implications for tumor progression and prognosis. *Cancer Sci.* **96**, 227–231 (2005). 15819721 10.1111/j.1349-7006.2005.00033.x PMC11158107

[17] Soong, R. et al. The expression of RUNX3 in colorectal cancer is associated with disease stage and patient outcome. *Br. J. Cancer*. **100**, 676–679 (2009). 19223906 10.1038/sj.bjc.6604899 PMC2653772

[18] Chen, X. et al. Loss of expression rather than cytoplasmic mislocalization of RUNX3 predicts worse outcome in non-small cell lung cancer. *Oncol. Lett.*10.3892/ol.2018.7993 (2018). 29545901 10.3892/ol.2018.7993 PMC5840764

[19] Berg, M. et al. Molecular subtypes in stage II-III colon cancer defined by genomic instability: early recurrence-risk associated with a high copy-number variation and loss of RUNX3 and CDKN2A. *PLoS ONE*. **10**, e0122391 (2015). 25879218 10.1371/journal.pone.0122391 PMC4399912

[20] Lotem, J. et al. Runx3 at the interface of immunity, inflammation and cancer. *Biochim. Biophys. Acta BBA - Rev. Cancer*. **1855**, 131–143 (2015). 10.1016/j.bbcan.2015.01.004 25641675

[21] Crowl, J. T. et al. Tissue-resident memory CD8 + T cells possess unique transcriptional, epigenetic and functional adaptations to different tissue environments. *Nat. Immunol.* **23**, 1121–1131 (2022). 35761084 10.1038/s41590-022-01229-8 PMC10041538

[22] Mami-Chouaib, F. et al. Resident memory T cells, critical components in tumor immunology. *J. Immunother Cancer*. **6**, 87 (2018). 30180905 10.1186/s40425-018-0399-6 PMC6122734

[23] Reis, B. S., Rogoz, A., Costa-Pinto, F. A., Taniuchi, I. & Mucida, D. Mutual expression of the transcription factors Runx3 and ThPOK regulates intestinal CD4 + T cell immunity. *Nat. Immunol.* **14**, 271–280 (2013). 23334789 10.1038/ni.2518 PMC3804366

[24] Selven, H. et al. High expression of IRS-1, RUNX3 and SMAD4 are positive prognostic factors in stage I-III colon cancer. *Cancers* **15**, 1448 (2023). 36900240 10.3390/cancers15051448 PMC10000923

[25] Selven, H., Busund, L. T. R., Andersen, S., Bremnes, R. M. & Kilvær, T. K. High expression of microRNA-126 relates to favorable prognosis for colon cancer patients. *Sci. Rep.* **11**, 9592 (2021). 33953222 10.1038/s41598-021-87985-3 PMC8100289

[26] Hald, S. M. et al. LAG-3 in Non–Small-cell lung cancer: expression in primary tumors and metastatic lymph nodes is associated with improved survival. *Clin. Lung Cancer*. **19**, 249–259e2 (2018). 29396238 10.1016/j.cllc.2017.12.001

[27] Bremnes, R. M. High-Throughput tissue microarray analysis used to evaluate biology and prognostic significance of the E-Cadherin pathway in Non-Small-Cell lung cancer. *J. Clin. Oncol.* **20**, 2417–2428 (2002). 12011119 10.1200/JCO.2002.08.159

[28] Bankhead, P. et al. QuPath: open source software for digital pathology image analysis. *Sci. Rep.* **7**, 1–7 (2017). 29203879 10.1038/s41598-017-17204-5 PMC5715110

[29] Schmidt, U., Weigert, M., Broaddus, C. & Myers, G. Cell detection with Star-Convex polygons. in Medical Image Computing and Computer Assisted Intervention – MICCAI 2018 (eds Frangi, A. F., Schnabel, J. A., Davatzikos, C., Alberola-López, C. & Fichtinger, G.) 11071 265–273 (Springer International Publishing, 2018).

[30] McShane, L. M. et al. REporting recommendations for tumor marker prognostic studies (REMARK). *Breast Cancer Res. Treat.* **100**, 229–235 (2006). 16932852 10.1007/s10549-006-9242-8

[31] Chen, X. et al. RUNX3/H3K27me3 co-expression defines a better prognosis in surgically resected stage I and postoperative chemotherapy-naive non-small-cell lung cancer. *J. Oncol.* 1–23 (2022). (2022). 10.1155/2022/5752263 PMC8970863 35368900

[32] Wu, Y. et al. Expression, clinical significance and correlation of RUNX3 and HER2 in colorectal cancer. *J. Gastrointest. Oncol.* **12**, 1577–1589 (2021). 34532112 10.21037/jgo-21-403 PMC8421900

[33] 33 Wang, C. et al. The heterogeneous immune landscape between lung adenocarcinoma and squamous carcinoma revealed by single-cell RNA sequencing. *Signal. Transduct. Target. Ther.* **7**, 289 (2022). 36008393 10.1038/s41392-022-01130-8 PMC9411197

[34] Hao, B. et al. Single-cell RNA sequencing analysis revealed cellular and molecular immune profiles in lung squamous cell carcinoma. *Transl Oncol.* **27**, 101568 (2023). 36270103 10.1016/j.tranon.2022.101568 PMC9586982

