# Supplementary material for: CD3+RUNX3+ lymphocyte density; an independent prognostic factor in colon and lung adenocarcinoma but not in lung squamous cell carcinoma
Source: Sci Rep. 2026 Feb 5;16:7361. doi: 10.1038/s41598-026-38765-4 (PMC12923705; doi:10.1038/s41598-026-38765-4)
Supplement: Supplementary file 1 — Supplementary Material 1 [file 41598_2026_38765_MOESM1_ESM.pdf]

SUPPLEMENTARY TABLES

Table S1: IHC protocol

Tissue Baking	Depar.	Antigen Retrieval	Block.	Primary Ab	Block.	Secondary Multimer	Amplify	Detection	Counterstain
SEQUENCE 1									
8 min 60°C	Disco. wash 3x12 min 68°C	CC1 40 min 95°C	Inhib 12 min 37°C AB block 16 min 37°C	Ab 1 Mo mono RUNX3 1:400 60 min 36°C	Post AB block 16 min 36°C	OMap anti-Mo HRP 16 min 37°C	AMP TSA HQ 8/8 min 37°C	Green HRP 32/16min 37°C	-
Stripping step: Denatruration CC2, 8 min, 100°C, Neutralize H2O2 Inhibitor, 20 min, 40°C									
SEQENCE 2									
-	-	-	-	Ab 2 Rb mono CD3 Predil. 40 min 36°C	-	OMap anti-Rb HRP 16 min 37°C	-	Purple/ H2O2 4/8 min 37°C	Hem II/ Bluing 4/4min 37°C

Table S2: Product references

Antibodies and Reagents	Reference	Company
RUNX3, 2B3 Ms monoclonal	Ab135248	abcam
CD3, 2GV6, Rb monoklonal	05278422001	Roche
Discovery Wash	7311079001	Roche
Ultra LCS (Liquid cover slip)	5424534001	Roche
Reaction buffer (10x)	5353955001	Roche
Discovery CC1 RUO	6414575001	Roche
Antibody Dilution buffer	5280524001	Roche
Benchmark Ultra CC2	5424542001	Roche
Discovery Inhibitor RUO	7017944001	Roche
Discovery Antibody block RUO	5268869001	Roche
Discovery AMP HQ kit RUO	6472320001	Roche
Discovery anti HQ HRP RUO	6442544001	Roche
OmniMap anti-Ms HRP RUO	5269652001	Roche
OmniMap anti-Rb HRP RUO	5269679001	Roche
Discovery Green HRP kit RUO	8478295001	Roche
Discovery Purple Kit RUO	7053983001	Roche
Hematoxylin II	5277965001	Roche
Bluing Reagent	5266769001	Roche
Ethanol 96%	20823.362	VWR, Avantor
Etnanol absolute	20821.296	VWR, Avantor
Xylene	28975.291	VWR, Avantor
Histokitt mounting medium	Assistant 1025/250	Sondheim/Rhoen

Table S3: Univariate analyses, of clinicopathological variables as prognosticators of DSS in COAD, LUAD and LUSC patients (log-rank test test, n = 452, 239 and 307)

	**COAD**					**LUAD**					**LUSC**				
	N(%)	5 Year	Median	HR(95%CI)	P	N(%)	5 Year	Median	HR(95%CI)	P	N(%)	5 Year	Median	HR(95%CI)	P
Age					0.013					0.650					0.931
≤65	110(24)	88	NA	1		115(48)	52	71	1		111(36)	64	235	1	
>65	342(76)	76	NA	1.83(1.21-2.77)		124(52)	52	NA	0.92(0.63-1.33)		196(64)	63	NA	1.02(0.69-1.49)	
Gender					0.388					0.027					0.095
Female	243(54)	78	NA	1		100(42)	59	190	1		77(25)	71	NA	1	
Male	209(46)	81	NA	0.85(0.58-1.23)		139(58)	46	57	1.54(1.06-2.23)		230(75)	61	235	1.48(0.97-2.26)	
Weightloss					0.012					0.849					0.917
<10%	248(55)	82	NA	1		221(92)	52	73	1		272(89)	64	235	1	
>10%	94(21)	68	NA	1.74(1.06-2.87)		18(8)	52	98	1.07(0.53-2.15)		34(11)	64	NA	0.97(0.51-1.84)	
Missing	110(24)					0					1(0)				
ECOG					0.424					0.049					0.099
1	237(52)	82	NA	1		153(64)	57	NA	1		168(55)	68	235	1	
2	149(33)	76	NA	1.31(0.86-1.99)		74(31)	43	51	1.54(1.02-2.32)		115(37)	59	114	1.51(1.01-2.26)	
3	54(12)	74	NA	1.18(0.62-2.28)		12(5)	38	31	1.86(0.67-5.21)		24(8)	59	NA	1.42(0.61-3.29)	
4	8(2)	50	47	2.31(0.31-16.98)											
Missing	4(1)														
pStage					<0.001					<0.001					0.000
1	72(16)	94	NA	1		110(46)	72	190	1		118(38)	75	235	1	
2	219(48)	89	NA	2.27(1.35-3.81)		63(26)	43	47	2.21(1.43-3.43)		121(39)	71	NA	1.39(0.92-2.08)	
3	161(36)	59	NA	8.04(4.58-14.11)		66(28)	22	25	3.79(2.29-6.25)		68(22)	30	16	4.39(2.49-7.74)	
Differentiation					<0.001					0.01					0.003
Well	36(8)	89	NA	1		48(20)	72	NA	1		33(11)	74	NA	1	
Moderate	329(73)	78	NA	1.47(0.76-2.82)		84(35)	50	57	2.17(1.33-3.56)		154(50)	70	235	1.44(0.78-2.66)	
Poor	75(17)	79	NA	1.45(0.67-3.15)		107(45)	44	50	2.6(1.62-4.17)		120(39)	52	71	2.53(1.33-4.81)	
Undifferentiated	4(1)	25	10	11.34(0.45-283.15)											
Missing	8(2)														
Vascular infiltration					<0.001					<0.001					0.010
No	199(44)	83	NA	1		202(85)	56	104	1		245(80)	67	235	1	
Yes	19(4)	45	30	5.22(1.66-16.37)		34(14)	24	30	2.16(1.19-3.94)		62(20)	49	39	1.76(1.05-2.94)	

Table S4: Associations between clinicopathological variables and high/low density (median cut-off) of different combinations of CD3 and RUNX3 expressing cells in COAD patients (N = 452, χ2 and Fisher's exact test as appropriate).

	CD3+RUNX3+/-			CD3+/-RUNX3+			CD3+RUNX3-			CD3+RUNX3+			CD3-RUNX3+		
	Low	High		Low	High		Low	High		Low	High		Low	High	
Age			0.886			0.440			0.587			0.219			0.710
≤65	50	52		55	47		54	48		57	45		49	53	
>65	155	152		150	157		151	156		148	159		156	151	
Gender			0.459			0.148			0.459			0.100			**0.015**
Female	117	108		105	120		117	108		104	121		100	125	
Male	88	96		100	84		88	96		101	83		105	79	
Weightloss			1.000			0.126			0.418			0.228			0.094
<10%	113	108		100	121		116	105		102	119		103	118	
≥10%	45	43		49	39		41	47		48	40		51	37	
ECOG			0.839			0.483			0.901			0.739			0.112
Normal	112	104		103	113		110	106		106	110		97	119	
Slightly reduced	63	70		68	65		66	67		67	66		75	58	
In bed <50%	26	24		28	22		23	27		26	24		27	23	
In bed >50%	3	4		5	2		4	3		5	2		5	2	
Site			0.115			**<0.001**			0.158			**0.001**			**0.021**
Right	93	108		85	116		100	101		86	115		91	110	
Transverse	27	32		25	34		27	32		25	34		27	32	
Left	12	5		10	7		13	4		11	6		6	11	
Sigmoid	72	58		84	46		64	66		82	48		79	51	
pStage			**0.028**			0.146			**0.027**			0.101			0.299
I	28	42		29	41		30	40		30	40		30	40	
II	92	101		95	98		89	104		92	101		96	97	
III	85	61		81	65		86	60		83	63		79	67	
Differentiation			0.227			0.058			0.431			0.168			**0.041**
Well	13	19		18	14		14	18		18	14		15	17	
Moderate	161	141		161	141		159	143		159	143		164	138	
Poor	27	38		24	41		28	37		25	40		24	41	
Undifferentiated	2	2		1	3		2	2		2	2		1	3	
Vasc+			0.072			0.331			0.403			0.127			0.378
No	90	85		86	89		89	86		84	91		87	88	
Yes	13	4		11	6		11	6		12	5		6	11	

Table S5: Associations between clinicopathological variables and high/low density (median cut-off) of different combinations of CD3 and RUNX3 expressing cells in LUAD patients (N = 239, χ2 and Fisher's exact test as appropriate).

	CD3+RUNX3+/-			CD3+/-RUNX3+			CD3+RUNX3-			CD3+RUNX3+			CD3-RUNX3+		
	Low	High		Low	High		Low	High		Low	High		Low	High	
Age			0.056			0.784			0.101			0.628			0.504
≤65	60	45		51	54		59	46		67	38		72	33	
>65	47	62		56	53		48	61		74	35		69	40	
Gender			0.579			0.052			0.267			0.531			0.072
Female	42	47		37	52		40	49		56	33		52	37	
Male	65	60		70	55		67	58		85	40		89	36	
Weight_loss			0.194			0.795			0.436			0.263			0.982
<10%	102	96		100	98		101	97		133	65		131	67	
≥10%	5	11		7	9		6	10		8	8		10	6	
Smoking			0.590			0.531			0.424			0.391			0.494
Never	5	6		6	5		5	6		9	2		6	5	
Present	63	69		62	70		62	70		83	49		85	47	
Previous	39	32		39	32		40	31		49	22		50	21	
ECOG			0.584			0.753			0.249			0.678			0.712
Normal	65	72		66	71		63	74		93	44		89	48	
Slightly reduced	35	30		35	30		36	29		41	24		45	20	
In bed <50%	7	5		6	6		8	4		7	5		7	5	
tStage			0.003			0.513			0.099			0.332			0.518
T1	30	46		33	43		33	43		44	32		46	30	
T2	40	43		45	38		39	44		59	24		59	24	
T3	18	14		16	16		19	13		22	10		20	12	
T4	19	4		13	10		16	7		16	7		16	7	
nStage			0.342			0.905			0.342			0.931			0.312
N0	70	75		71	74		70	75		95	50		93	52	
N1	23	15		20	18		23	15		26	12		29	9	
N2	14	17		16	15		14	17		20	11		19	12	
pStage			**0.043**			0.670			0.254			0.783			0.990
I	39	57		45	51		42	54		61	35		63	33	
II	34	27		33	28		34	27		42	19		40	21	
III	34	23		29	28		31	26		38	19		38	19	
Differentiation			0.355			0.116			0.856			0.756			0.185
Poor	49	50		50	49		51	48		66	33		68	31	
Moderate	41	33		42	32		37	37		50	24		51	23	
Well	17	24		15	26		19	22		25	16		22	19	
Vasc+			0.454			0.142			0.719			0.427			0.427
No	88	92		87	93		89	91		117	63		117	63	
Yes	18	13		20	11		17	14		23	8		23	8	

Table S6: Associations between clinicopathological variables and high/low density (median cut-off) of different combinations of CD3 and RUNX3 expressing cells in LUSC patients (N = 307, χ2 and Fisher's exact test as appropriate).

	CD3+RUNX3+/-			CD3+/-RUNX3+			CD3+RUNX3-			CD3+RUNX3+			CD3-RUNX3+		
	Low	High		Low	High		Low	High		Low	High		Low	High	
Age			0.319			0.619			0.619			0.455			0.803
≤65	47	56		54	49		49	54		48	55		53	50	
>65	91	82		84	89		89	84		90	83		85	88	
Gender			0.585			0.056			0.413			0.101			**0.029**
Female	34	39		29	44		33	40		30	43		28	45	
Male	104	99		109	94		105	98		108	95		110	93	
Weight_loss			0.687			0.719			0.433			0.459			0.719
<10%	120	124		121	123		119	125		120	124		121	123	
≥10%	17	14		17	14		18	13		18	13		17	14	
Smoking			0.688			0.942			0.687			0.829			0.833
Never	4	3		4	3		4	3		4	3		4	3	
Present	88	95		92	91		88	95		93	90		93	90	
Previous	46	40		42	44		46	40		41	45		41	45	
ECOG			**0.031**			0.783			0.119			0.783			0.471
Normal	68	85		78	75		70	83		78	75		81	72	
Slightly reduced	54	47		48	53		53	48		48	53		48	53	
In bed <50%	16	6		12	10		15	7		12	10		9	13	
tStage			0.446			0.099			0.359			0.057			**0.033**
T1	40	40		31	49		40	40		32	48		29	51	
T2	45	55		52	48		44	56		48	52		57	43	
T3	35	25		34	26		35	25		36	24		33	27	
T4	18	18		21	15		19	17		22	14		19	17	
nStage			0.564			0.763			0.564			0.858			0.502
N0	93	101		96	98		93	101		98	96		97	97	
N1	37	31		36	32		37	31		34	34		36	32	
N2	8	6		6	8		8	6		6	8		5	9	
pStage			0.664			0.173			0.510			0.238			0.469
I	52	53		45	60		51	54		46	59		48	57	
II	52	57		60	49		52	57		57	52		59	50	
III	34	28		33	29		35	27		35	27		31	31	
Differentiation			0.697			0.928			0.140			0.590			0.556
Poor	54	53		53	54		53	54		50	57		57	50	
Moderate	67	72		69	70		65	74		71	68		65	74	
Well	17	13		16	14		20	10		17	13		16	14	
Vasc+			0.137			0.372			0.372			0.372			0.234
No	104	115		113	106		106	113		113	106		114	105	
Yes	34	23		25	32		32	25		25	32		24	33	

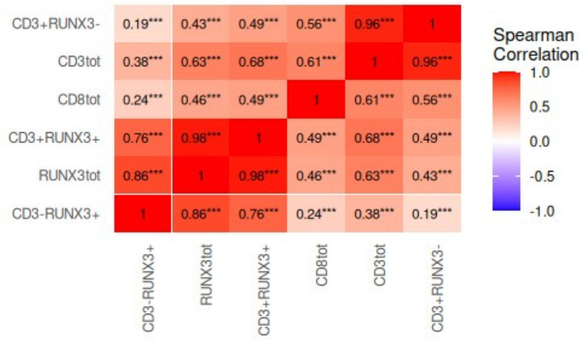

Figure S1: Correlations between immune markers for COAD patients.

*P-values: * <0.05, ** <0.01, ***<0.001*

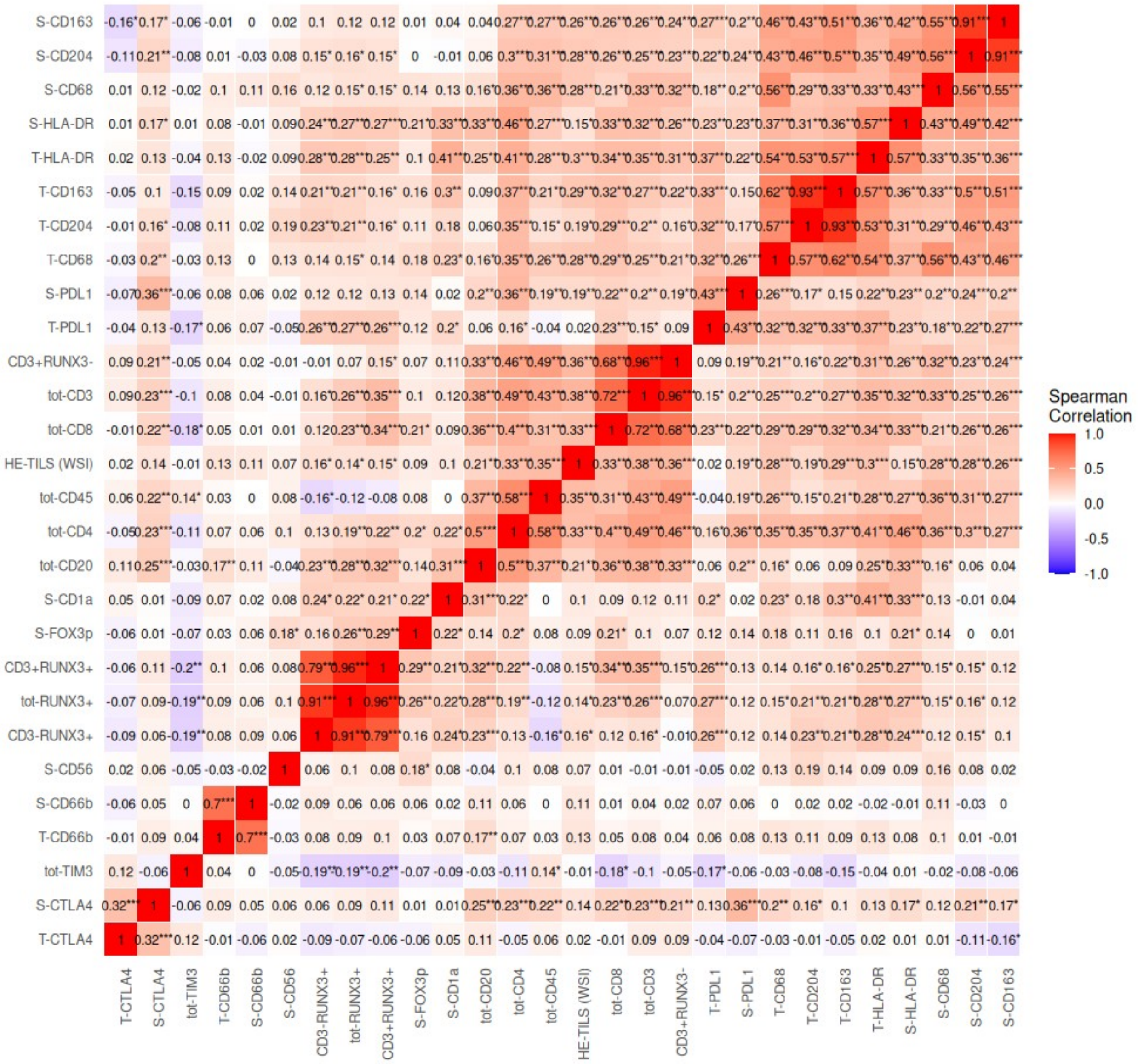

Figure S2: Correlations between immune markers for LUAD patients. “T” and “S” indicates that the marker was evaluated in Tumor and/or Stroma separately while tot values indicate that no discrimination between tumor and stroma was conducted.

*P-values: * <0.05, ** <0.01, ***<0.001*

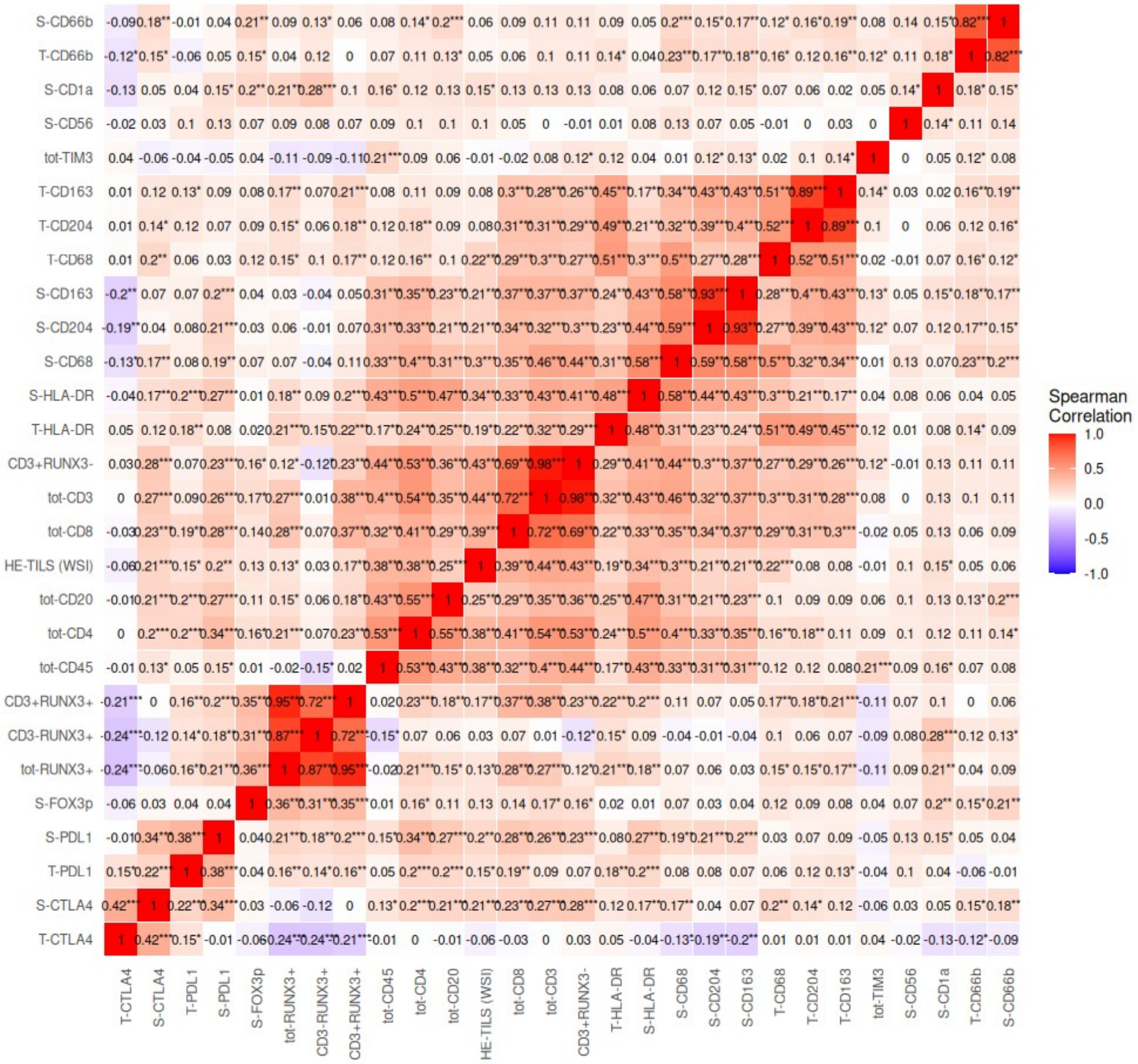

Figure S3: Correlations between immune markers for LUSC patients. "T" and "S" indicates that the marker was evaluated in Tumor and/or Stroma separately while tot values indicate that no discrimination between tumor and stroma was conducted.

*P values: * <0.05, ** <0.01, ***<0.001*

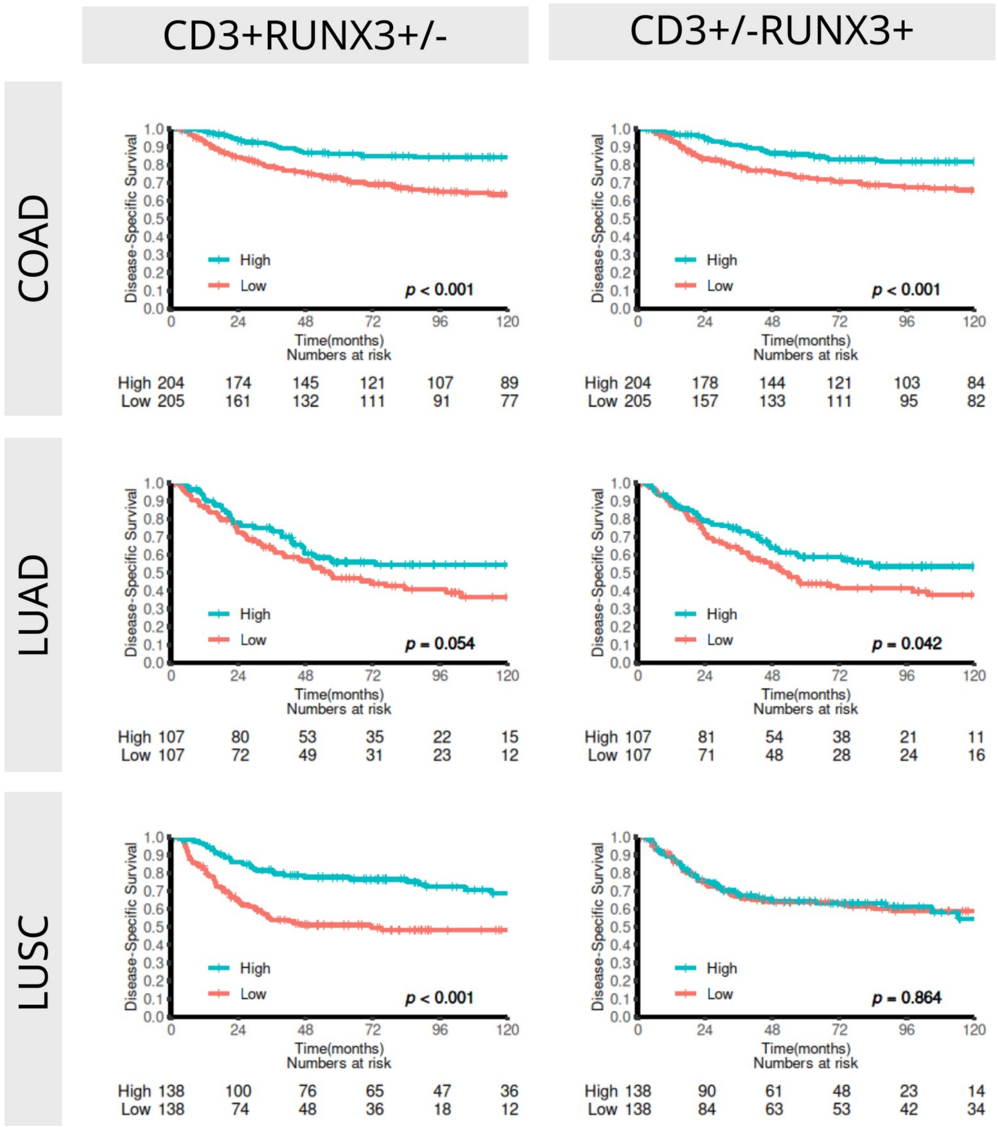

Figure S4: Disease-specific survival curves for overall CD3 and RUNX3 densities in COAD (n = 452), LUAD (n = 239) and LUSC (n =307) using median cut-offs.

Example script to replicate our QuPath pipeline

```
// Author: Thomas Kilvaer
// Email: thomas.k.kilvar@uit.no

import qupath.ext.stardist.StarDist2D
import qupath.lib.scripting.QP

QP.setColorDeconvolutionStains('{"Name" : "H-DAB modified", "Stain 1" : "Hematoxylin",
"Values 1" : "0.86297 0.49199 0.115", "Stain 2" : "CD3", "Values 2" : "0.28107 0.94625 0.16004",
"Stain 3" : "RUNx3", "Values 3" : "0.85725 0.23107 0.46014", "Background" : " 255 255 255"}');
// Color deconvolution has to be set manually according to staining specifics – these values work
for us
QP.selectCells()
QP.clearSelectedObjects()
QP.selectTMACores()
createAnnotationsFromPixelClassifier("thresholder", 10000.0, 1000.0, "DELETE_EXISTING",
"INCLUDE_IGNORED") // A reasonable thresholder must be made to exclude any empty regions

def modelPath = "path_to_stardist_model" // Model can be obtained upon request to the author

// Get current image - assumed to have color deconvolution stains set
var imageData = QP.getCurrentImageData()
var stains = imageData.getColorDeconvolutionStains()

def stardist = StarDist2D
    .builder(modelPath)
    .layout("BYXC")
    .preprocess( // Extra preprocessing steps, applied sequentially
        ImageOps.Channels.deconvolve(stains),          // Color deconvolution
        ImageOps.Channels.extract(0,2),                // Extract stains (indexing starts at 0)
        ImageOps.Channels.maximum(),                   // Only use the max value of either stain
        ImageOps.Core.clip(0.05, 0.4),                 // Filter out extreme values
        ImageOps.Filters.median(2)                     // Apply a median filter with radius of 2
        )
    .threshold(0.5)                                    // Probability (detection) threshold
    .keepClassifiedBackground(false)
    .pixelSize(0.5)                                    // Resolution for detection
    .tileSize(256)
    .cellExpansion(2)                                   // Expand nuclei to approximate cell
                                                       boundaries
    .measureShape()                                    // Add shape measurements
    .measureIntensity()                                // Add cell measurements (in all compartments)
    .build()


def pathObjects = QP.getAnnotationObjects()

// Run detection for the selected objects
if (pathObjects.isEmpty()) {
    QP.getLogger().error("No parent objects are selected!")
    return
}
```

```
stardist.detectObjects(imageData, pathObjects)
stardist.close() // This can help clean up & regain memory
println('Done!')

QP.selectCells();
QP.runPlugin('qupath.lib.algorithms.IntensityFeaturesPlugin',
'{"pixelSizeMicrons":2.0,"region":"ROI","tileSizeMicrons":25.0,"colorOD":true,"colorStain1":true,
"colorStain2":true,"colorStain3":true,"colorRed":true,"colorGreen":true,"colorBlue":true,"colorHue
":true,"colorSaturation":true,"colorBrightness":true,"doMean":false,"doStdDev":true,"doMinMax":f
alse,"doMedian":true,"doHaralick":true,"haralickDistance":1,"haralickBins":32}')

runObjectClassifier("cell_classifier"); // A reasonable cell classifier has to be made
```
